# Efficiency enhancement of Cu_2_ZnSnS_4_ solar cells via surface treatment engineering

**DOI:** 10.1098/rsos.171163

**Published:** 2018-01-03

**Authors:** Rongrong Chen, Jiandong Fan, Hongliang Li, Chong Liu, Yaohua Mai

**Affiliations:** 1 College of Physics Science and Technology, Hebei University, Baoding 071002, People's Republic of China; 2 Institute of New Energy Technology, College of Information Science and Technology, Jinan University, Guangzhou 510632, People's Republic of China

**Keywords:** Cu_2_ZnSnS_4_, sol–gel method, etching, sulfurization

## Abstract

Pure-sulphide Cu_2_ZnSnS_4_ (CZTS) thin film solar cells were prepared by a low-cost, non-toxic and high-throughput method based on the thermal decomposition and reaction of sol–gel precursor solution, followed by a high temperature sulfurization process in sulphur atmosphere, which usually gave rise to the unexpected Cu-poor and Zn-rich phase after sulfurization. In order to remove the formation of detrimental secondary phases, e.g. ZnS, a novel method with hydrochloric acid solution treatment to the CZTS absorber layer surface was employed. By using this method, a competitive power conversion efficiency as high as 4.73% was obtained, which is a factor of 4.8 of that of the control CZTS solar cell without surface treatment. This presents a customized process for CZTS photovoltaic technologies that is more environmentally friendly and considerably less toxic than the widely used KCN etching approach.

## 1. Introduction

At present, thin film solar cells such as copper indium gallium selenium solar cells have received considerable attention because of their simple preparation technology, large-scale production and higher photoelectric conversion efficiency [1–3]. Nevertheless, indium and gallium are rare in the earth and rather expensive, which may limit its large-scale application in the future. Copper zinc tin sulphur (CZTS) thin film solar cells with a relatively cheap and abundant zinc and tin element, instead of indium and gallium, significantly reduced the costs and are found to be more suitable for the large-scale application [4–8]. In addition, as an important compound thin film absorber material, CZTS exhibits superior optical and electronic properties, as well as a suitable band gap (approx. 1.5 eV). It is well known that CZTS has a great light absorption coefficient of more than 10^4 ^cm^−1^ in the visible light region, and its theoretical power conversion efficiency (PCE) is more than 30% [9–11]. So far, various methods have been employed to prepare CZTS film, such as sputtering [12], thermal evaporation [13], electrodeposition [14], nanoparticles [15] and hydrazine solution [16]. Usually, these methods use high vacuum deposition systems or employ toxic chemicals. Here, we adopt a kind of low-cost, high-throughput and non-toxic method to produce CZTS thin film and the post-sulfurization to obtain the absorber thin film with high crystallization [17–19]. CZTS film with Cu-poor and Zn-rich phase has been proved to have superior photoelectric performance [20–22]. However, the presence of excessive zinc will induce the formation of the ZnS binary phase after sulfurization. The surface treatment has been carried out to remove Cu_2−*x*_S by using potassium cyanide (KCN) [23–25]. Likewise, Fairbrother *et al.* [26] developed a selective chemical etch with hydrochloric acid (HCl) to remove the detrimental secondary phases of CZTS thin film prepared by DC-magnetron sputtering technique. Nevertheless, as far as we know, there is no effective method to remove the ZnS phase of CZTS absorber layer prepared via thermal decomposition and reaction of sol–gel solution process, which might be the key reason that the corresponding solar cells are still limited by their inferior photovoltaic performance.

Herein, a new non-toxic route with a certain concentration of hydrochloric acid (HCl) solution was employed to ensure a thorough removal of ZnS binary phase. In particular, we carefully explored the effect of different concentrations and etching time of hydrochloric acid solution and immersion time on the CZTS surface morphology and solar cell photovoltaic performances. Such CZTS solar cell with the further modification process of immersing in 5% v/v HCl solution for 300 s at 75°C allowed to obtain the highest PCE of 4.73%, which gave rise to the improved PCE by a factor of 4.3.

## 2. Material and methods

### 2.1. Cu_2_ZnSnS_4_ thin film preparation

**Figure 1.**
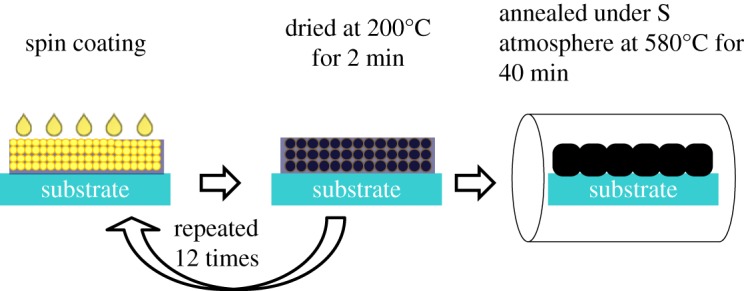
Schematic of the formation of the CZTS thin films by the sol–gel route.

All reagents were of analytical grade and used without any further purification. The CZTS precursor solutions were made by adding 0.44 M cupric chloride dihydrate (CuCl_2_·2H_2_O, 99%), 0.25 M tin dichloride dihydrate (SnCl_2_·2H_2_O, 98%), 0.3 M zinc chloride (ZnCl_2_, 98%) and 1.5 M thiourea (CH_4_N_2_S, 99%) into 10 ml dimethylsulfoxide (DMSO, anhydrous) at room temperature. The precursor composition of metal salt was Cu/(Zn + Sn)  = 0.8 and Zn/Sn = 1.2. The precursor solution was deposited via spin-coating onto Mo-coated soda lime glass substrates and then placed on a 200°C preheated hot plate for 2 min. This coating–drying cycle was repeated 12 times to get the desired thickness of CZTS precursors film. After that, the prepared precursors were annealed at 580°C for 40 min inside a graphite box in a tube furnace of sulphur powders (150 mg) with N_2_ atmosphere under 0.05 MPa during sulfurization as shown in figure 1. After sulfurization, the absorbers were immersed in HCl solution of different HCl concentrations (0–10% v/v) and etch time (0–600 s) at 75°C in order to remove zinc-rich phases and clean the surface from contaminations and oxides.

### 2.2. Cu_2_ZnSnS_4_ solar cell devices fabrication

For solar cell fabrication, an approximately 80 nm thick CdS buffer layer was deposited by chemical bath deposition, and 80 nm of i-ZnO followed immediately by 350 nm of ITO layer were deposited by RF magnetron sputtering. Finally, Al was thermally evaporated on ITO layer to form top contact fingers via shadow mask. Each device has a total area of approximately 0.47 cm^2^ defined by mechanical scribing.

### 2.3. Characterization and analysis

The crystal structure was characterized by Bruker D8 Advance X-ray diffractometer (XRD) with Cu K*α* radiation at 40 kV and 40 mA. Field-emission scanning electron microscopy (SEM) was used to characterize the morphology of the obtained thin film. Both top-down and cross-sectional views were obtained using a JEOL JSM-7500F. Current–voltage (*J*–*V*) characteristics of CZTS solar cells were measured using a semiconductor device analyser (Keithley 2601B) and a SAN-EI solar simulator (XES-100S1) with an AM 1.5 G spectrum. The illumination power on the sample was adjusted to 1000 W m^−2^ using a certified reference solar cell (RS-ID-4). The scan rate was fixed to 0.15 V s^−1^. Raman scattering spectroscopy was performed using a LabRAM HR evolution of Horiba Raman scattering system with a 100× magnification lens and in the backscattering configuration. Raman scattering measurements were performed using excitation wavelength of 532 and 325 nm.

## 3. Results and discussion

**Figure 2.**
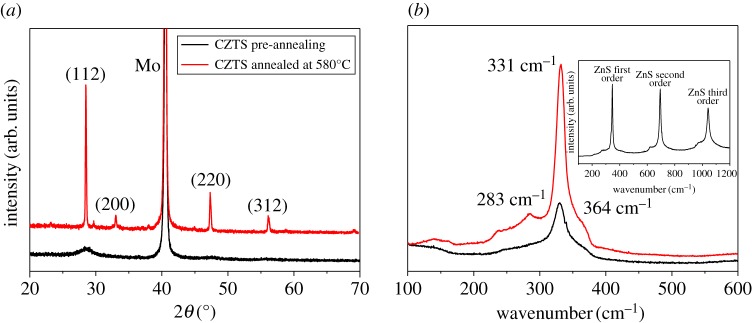
(*a*) XRD patterns of CZTS thin films before and after sulfurization. (*b*) Raman spectra of CZTS thin films before and after sulfurization. Inset is the Raman spectra of ZnS binary phase.

**Figure 3.**
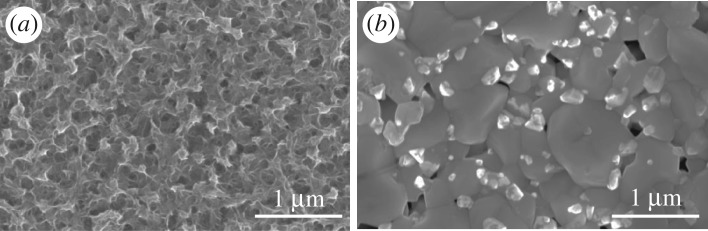
(*a,b*) are SEM top-down view of the CZTS thin films before and after sulfurization, respectively.

Figure 2*a* shows the diffraction peaks of CZTS film located at 28.4°, 33.0°, 47.3° and 56.1° that correspond to the (112), (200), (220) and (312) planes of kesterite crystal structure (PDF#26–0575), respectively. The weak peak of unannealed CZTS film indicated by a black line in the figure is ascribed to the weaker crystallization. High and sharp peaks are observed after sulfurization at high temperature due to the improved film crystallinity and grain growth. To study the impact of sulfurization on the structural properties, Raman spectroscopy measurements have also been carried out on the CZTS film before and after sulfurization modification. Figure 2*b* shows the Raman spectra measured with an excitation wavelength of 532 nm on the surface of the CZTS film. The spectra are characterized by the presence of main peaks at 331 cm^−1^ identified as the main peaks A1 vibration mode of CZTS [27–29], as well as weaker CZTS characteristic peaks at about 283 and 364 cm^−1^, respectively. The peak intensity is enhanced after sulfurization treatment, which is consistent with the XRD results. It is worthy of noting that the ZnS binary phase can be detected with an excitation wavelength of 325 nm (inset in figure 2*b*), which is favourable to the study of removal of the ZnS binary phase. Before and after sulfurization process, CZTS absorber morphology are shown in the SEM image in figure 3*a,b*. From the SEM top-down view image, one can find that the CZTS film was compact and uniform but poorly crystalline before sulfurization. After sulfurization treatment, the crystallinity of CZTS thin film was improved and the average crystal grain was approximately 1 µm. Likewise, there are many small and light particles embedded in the grain boundaries after sulfurization process.

**Figure 4.**
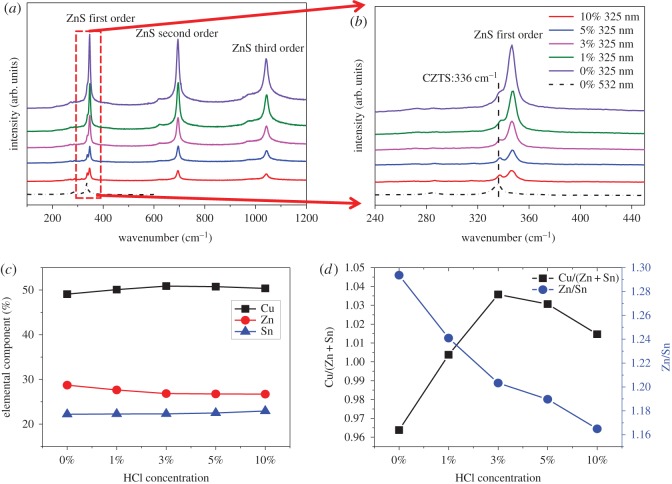
(*a*) Raman spectra with excitation wavelength of 325 and 532 nm taken for the as-grown sample etched with different concentrations solution of HCl at 75°C for 300 s; (*b*) corresponding enlarged Raman spectra of (*a*); (*c,d*) the evolution of relative cation composition after etching the CZTS film with different HCl concentrations at 75°C for 300 s that derived from EDS data.

**Figure 5.**
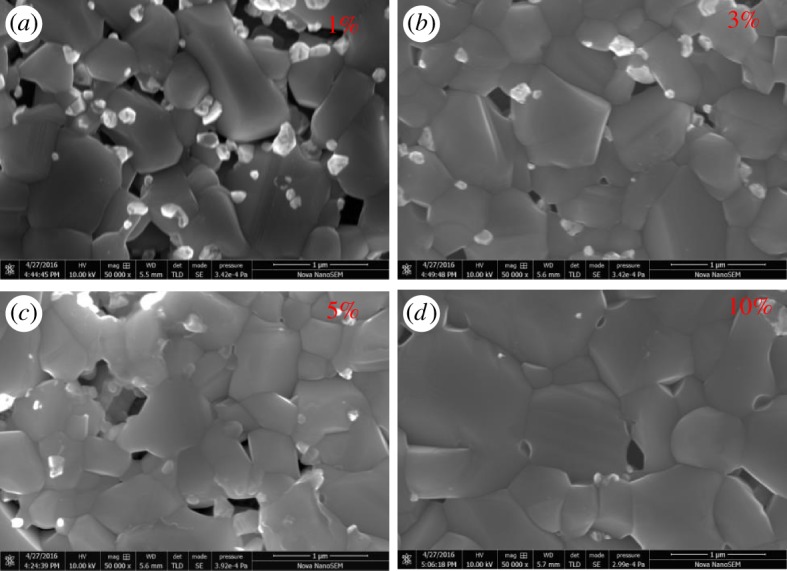
Top-down SEM images of CZTS thin films at (*a*) 1%, (*b*) 3% (*c*) 5% and (*d*) 10% concentrations HCl solution soaking for 300 s.

As mentioned previously, the XRD and Raman with an excitation wavelength of 532 nm cannot detect the ZnS binary phase, the laser with the wavelength of 325 nm was adopted to further study the CZTS structure. As already indicated, these excitation conditions allow excitation of the main ZnS vibrational modes. As shown in figure 4*a,b*, the presence of ZnS in the surface region of the non-etched CZTS film was corroborated by the detection in Raman spectrum of first-order (around 348 cm^−1^), second-order (around 696 cm^−1^) and third-order (around 1044 cm^−1^), respectively [30,31]. This demonstrated that the thin film surface has ZnS binary phase, which is associated with the small particles. By means of HCl etching for 300 s under 75°C with the concentration of hydrochloric acid increasing from 0% to 10%, the ZnS peaks were demonstrated to be drastically reduced. And in the case of 10% concentration etching, the ZnS Raman peaks were reduced even more. At the same time, it can be seen that the CZTS peak was relatively enhanced, which suggested that HCl solution can effectively remove the ZnS on the surface of CZTS film. Figure 4*c,d* shows the change of element content by means of the EDS results of CZTS thin film etching by HCl solution at different volume ratio. Clearly, the Zn/Sn ratio decreased with the HCl treatment in the case of 10% concentration, where its value was close to 1.16. Firstly, the Cu/(Zn+Sn) ratio increased with HCl concentration, which could be attributed to the diminution of the Zn concentration. When the HCl concentration increased up to 5%, the Cu/(Zn + Sn) ratio reduced with HCl concentration, which could be associated with the diminution of the Cu concentration. Again, these proved that HCl solution can effectively remove the ZnS binary phase presented on the surface of CZTS film. As mentioned above, figure 3*b* shows that the non-etched CZTS films have many small crystals with the size of approximately 100 nm, which proved the presence of the ZnS binary phase. Figure 5 shows the top-down SEM images of the CZTS thin film etching by HCl solution with a different volume ratio. After the etching process with HCl-based solution, an obvious reduction of the small crystals on the surface of CZTS thin film was clearly seen. Further increasing the HCl concentration shows a drastic decrease of the small crystals on the CZTS surface, which could be explained by the following chemical reaction:

3.1 $$\text{ZnS}+\text{HCl}\to {\text{ZnCl}}_{2}+{\text{H}}_{2}\text{S}↑$$

We further studied the effect of etching time on the structure and composition of CZTS thin films (electronic supplementary material, figure S1). Here, we fixed the concentration of HCl as 0.5% according to the optimized results above. As shown in electronic supplementary material, figure S1a, the samples prepared with five different etching times have three strong Raman peaks at 346, 694 and 1041 cm^−1^, respectively. Again, these peaks corresponded to the first-order, second-order and third-order characteristic Raman peaks of ZnS. Owing to the strong Raman vibration peak intensity of ZnS, the characteristic Raman peak at 336 cm^−1^ at CZTS almost could not be clearly found, which was similar to the result provided in the previous section. With the increase of the etching time of hydrochloric acid, the characteristic peak intensity of ZnS gradually decreased, indicating that the longer etching time of hydrochloric acid, the stronger ability of ZnS to etch the surface of the film. Similarly, we amplified the Raman spectrum near the Raman peak of the first-order characteristic of ZnS as shown in electronic supplementary material, figure S1b. In the case of non-etching, we can see the characteristic Raman peaks of CZTS at 336 cm^−1^. Gradually, when the hydrochloric acid etching time increases, the peak intensity of CZTS displayed relative enhancement while the peak intensity of ZnS decreased. Afterwards, we explored the etching time-induced change of CZTS composition, as shown in electronic supplementary material, figure S1c and S1d, with the increase of the etching time, the Zn content decreased gradually and the ratio of Zn/S decreased from 1.30 to 1.2, which indicated that the longer the etching time, while keeping the same concentration of hydrochloric acid solution, the more ZnS was removed, which was consistent with the previous study. Electronic supplementary material, figure S2 displays the SEM images of CZTS thin film with different hydrochloric acid etching time. Again, after the surface of the film was etched, the white bright spot on the surface of the film was reduced, indicating that the HCl etching modification on the CZTS surface was effective to remove the ZnS binary phase. Comparably, there was not too much ZnS secondary phase in the bulk both before and after etching in comparison to the surface of CZTS thin film (electronic supplementary material, figure S3). In this scenario, it appears that the HCl mainly etched the surface of kesterite film and thereafter removed the ZnS secondary phase. Likewise, the crystallization orientation and crystal quality of CZTS absorber layer were obviously improved thanks to the optimized phase and stoichiometric ratio after HCl etching process.

**Figure 6.**
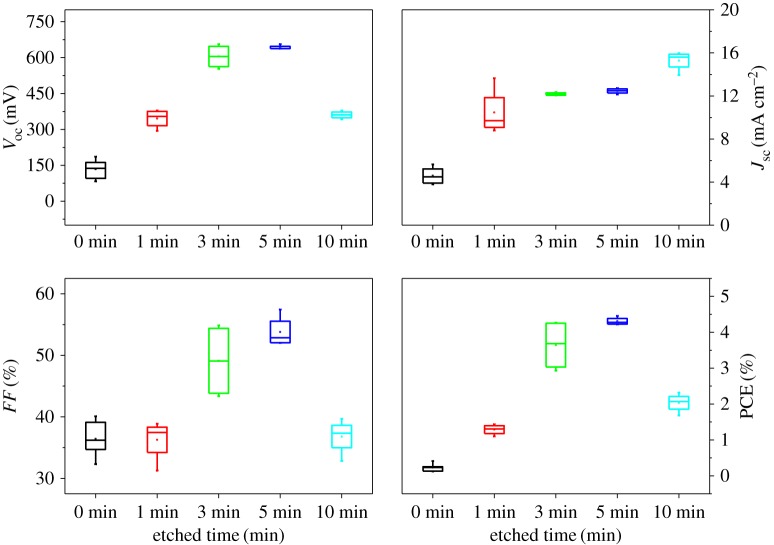
Photovoltaic performance of CZTS solar cell etching with different time.

An additional fact concerning the etching process via presented HCl solution was proved by its effect on the photovoltaic parameters of the obtained CZTS solar cells. Electronic supplementary material, figure S4 exhibits the illuminated *J−V* characteristics of the solar cells prepared with different HCl solution concentration. The conversion efficiency of non-etched CZTS solar cell was only 0.5%. After the HCl solution etching with the concentration of 5% v/v, the conversion efficiency was improved greatly up to 2.4%, which gave rise to improved PCE by a factor of five times. Further, the photovoltaic parameters of CZTS solar cell were demonstrated to be significantly reduced when the HCl solution with the concentration of 10% v/v was applied to the samples, yielding the solar cells with only 0.7% conversion efficiency. The reason may be ascribed to the high concentration of HCl solution that is harmful for the CZTS absorber. In the case of etching time, when the etching time is increased from 0 to 5 min, the open circuit voltage (*V*_OC_) and the short circuit current (*J*_SC_) increased with the increase in the etching time, so the PCE was also increasing. The maximum PCE was 4.73% when the etching time was 5 min (figure 6). However, when the etching time of hydrochloric acid increased to 10 min, the *V*_OC_ and filling factor decreased, and the corresponding PCE began to decrease. The reason should be that the etching time was too long and the hydrochloric acid destroyed the CZTS film. It is necessary to select the appropriate etching time while etching the binary phase ZnS on the surface of CZTS film.

## 4. Conclusion

In summary, an approach regarding hydrochloric acid solution etching Cu_2_ZnSnS_4_ (CZTS) absorber layer surface was reported. The results demonstrated that the hydrochloric acid solution with certain concentration and etching time can efficiently remove the zinc-rich phases and afterward clean the surface from contaminations and oxides. Such etched CZTS solar cell with 5% v/v HCl concentration for 300 s at 75°C allowed to obtain the highest PCE of 4.73%, which gave rise to improved PCE by a factor of 4.8 time. Further studies toward higher efficiency, e.g. post-selenization of precursor film and varying the [S]/([S] + [Se]) ratios via band gap engineering by means of optimizing the selenization condition, is underway.

## Supplementary Material

Rongrong Chen figures ESM

## Data accessibility

All the relevant data are presented in this paper and in the electronic supplementary material.
