# Supplementary material for: Efficiency enhancement of Cu2ZnSnS4 solar cells via surface treatment engineering
Source: R Soc Open Sci. 2018 Jan 3;5(1):171163. doi: 10.1098/rsos.171163 (PMC5792906; doi:10.1098/rsos.171163)
Supplement: Rongrong Chen figures ESM [file rsos171163supp1.docx]

**Electronic Supplementary Information**

**Efficiency Enhancement of Cu_2_ZnSnS_4_ Solar Cells *via* Surface Treatment Engineering**

Rongrong Chen,^a^ Jiandong Fan,*^ab^ Hongliang Li, ^a^ Chong Liu ^b^ and Yaohua Mai*^ab^

^a^*College of Physics Science and Technology, Hebei University, Baoding, 071002 China*

^b^*Institute of New Energy Technology, College of Information Science and Technology, Jinan University, Guangzhou 510632, China*

*Author for correspondence (E-mail: (J. F.) jdfan@jnu.edu.cn; E-mail: (Y. M.) yaohuamai@jnu.edu.cn).

Figure S1. (a) Raman spectra with excitation wavelength of 325 nm and 532nm taken for the as-grown sample etched with different etching time; (b) corresponding enlarged Raman spectra of (a); (c) and (d) the evolution of relative cation composition after etching the CZTS film with different HCl concentrations at 75 °C for 300 s that derived from EDS data.

Figure S2. SEM images of CZTS thin film with different HCl etching time: (a) 0 min (b) 1 min (c) 3 min (d) 5 min

Figure S3 Cross-sectional SEM images of CZTS solar cells before and after etching

Figure S4. The photovoltaic performances of CZTS solar cell etched with different HCl concentration.
